# Fine-mapping using the weighted average method for a case-control study

**DOI:** 10.1186/1471-2156-6-S1-S67

**Published:** 2005-12-30

**Authors:** Kijoung Song, Mohammed S Orloff, Qing Lu, Robert C Elston

**Affiliations:** 1 Department of Epidemiology and Biostatistics, Case Western Reserve University, Cleveland, OH, USA

## Abstract

We present a new method for fine-mapping a disease susceptibility locus using a case-control design. The new method, termed the weighted average (WA) statistic, averages the Cochran-Armitage (CA) trend test statistic and the difference between the Hardy-Weinberg disequilibrium test statistic for cases and controls (the HWD trend). The main characteristics of the WA statistic are that it improves on the weaknesses, and maintains the strengths, of both the CA trend test and the HWD trend test. Data from three different populations in the Genetic Analysis Workshop 14 (GAW14) simulated dataset (Aipotu, Karangar, and Danacaa) were first subjected to model-free linkage analysis to find regions exhibiting linkage. Then, for fine-scale mapping, 140 SNPs within the significant linkage regions were analyzed with the WA test statistic on replicates of the three populations, both separately and combined. The regions that were significant in the multipoint linkage analysis were also significant in this fine-scale mapping. The most significant regions that were obtained using the WA statistic were regions in chromosome 3 (B03T3056–B03T3058, *p*-value < 1 × 10^-10 ^) and chromosome 9 (B09T8332–B09T8334, *p*-value 1 × 10^-6 ^). Based on the results of the simulated GAW14 data, the WA test statistic showed good performance and could narrow down the region containing the susceptibility locus. However, the strength of the signal depends on both the strength of the linkage disequilibrium and the heterozygosity of the linked marker.

## Background

It has been shown that fine-scale mapping of a susceptibility locus for a complex disease can be accomplished by evaluating the deviation from Hardy-Weinberg equilibrium (HWE). For example, Feder et al. [1], Nielsen et al. [2] and Jiang et al. [3] have discussed using the Hardy-Weinberg disequilibrium (HWD) test on affected individuals alone. From their results, this HWD test tends to perform well for a recessive disease model and could be more precise in gene localization, but has no power at all for a multiplicative disease model. For case-control studies, Sasieni et al. [4] showed that the Cochran-Armitage (CA) trend test, which uses genotype data, is more appropriate than the allele-based test when HWE is violated. The CA trend test is good when there is dominant inheritance, has power where the HWD test has no power, but requires allowance for population structure. Devlin and Roeder [5] proposed genomic control to allow for population heterogeneity when using the CA trend test. Song and Elston [6] proposed the HWD trend test, which compares the squared difference in HWD between cases and controls, divided by its estimated variance, with the chi-square distribution with 1 d.f. They showed by simulation that the HWD trend test statistic is not inflated by population stratification.

Song and Elston [6] developed a weighted average (WA) statistic that mitigates against the weaknesses and maintains the strong points of both the CA trend test and the HWD trend test. In this study, we apply model-free linkage analysis to find regions of interest, and then the WA statistic method to fine map, the Genetic Analysis Workshop (GAW14) simulated dataset in order to find susceptibility disease genes. Finally, we compare the results of the WA test with those of the CA trend and the HWD trend tests and find that for these data the WA is virtually identical to the CA test.

## Methods

For a case-control study with a diallelic marker locus, write the CA trend test statistic as  and the HWD trend test statistic as . Then the WA statistic is given by



where 

Song and Elston [6] used simulation to show that asymptotically the random variable *Y *is well approximated by a Gamma distribution, *F*(*y*; *θ*, *κ*), with mean *μ *= 1.78 and variance *σ*^2 ^= 3.45, i.e., *θ *= *σ*^2 ^/*μ *= 1.94 and *κ *= *μ*^2 ^/*σ*^2 ^= 0.92. The best value to take for *w *was problematic because it depends on details of the alternate hypothesis, which are usually unknown. Thus, they chose the value of *w *indicated above after performing several exploratory simulation studies. They modeled the empirical *α *(*p*-value) that would predict the probability of type I error from the *α *corresponding to 1 - *F*(*y*_(1-*α*)_; *θ*, *κ*) using the regression equation



where *α*_*i *_(*i *= 1, 2, ..., *n*) was estimated from a series of *n *simulation experiments. Based on extensive simulation results for various sample sizes > 50, they estimated *a *to be

1.4922[1/log_*e *_*R *× log_*e *_*S*] + 0.1208[log_*e *_(*R*/*S*)] - 1.6929[1/(log_*e *_*R *× log_*e *_*S *× (0.5 - |*M *- 0.5|))] + 0.9250(*M *- 0.5)^2 ^

when there are R cases and S controls and different values of the marker allele frequency M. To adjust the test statistic by a factor that measures the amount of variance inflation caused by population stratification and cryptic relatedness, the variance of  can be adjusted using the genomic control method of Devlin and Roeder [5] when the inflation factor is >1.

### Genome scan

We analyzed the binary trait affected/unaffected in replicates from the three different populations in the dataset (Aipotu, Karangar, and Danacaa). For each of the three different populations, we first used the microsatellite markers that were on average 7.5 cM apart to perform a linkage scan. We used SIBPAL (S.A.G.E., version 4.6), a model-free linkage program, to perform a multipoint linkage analysis. Evidence for linkage was evaluated by Haseman-Elston regression [7] using a weighted average of the squared trait difference and the squared mean-corrected trait sum (option W4 [8]). The empirical *p*-values for each marker location were calculated and pooled over the populations.

### Fine mapping

After reviewing the first-stage genome-scan linkage results, we selected for a case-control study 140 SNPs 0.3 cM apart that encompassed the significant linkage regions. From each of three replicates (one from each population), we randomly sampled 100 affected probands as our cases and 100 unaffected probands as our controls. In an attempt to induce population stratification, we also pooled the replicates from the three populations to obtain 300 cases and 300 controls. Then the WA test statistic was calculated for each of the four different samples using the selected SNPs. We repeated the sampling from each population 5 times and recorded the percentage of times that the hypothesis of no disequilibrium was rejected. The potential confounding effect of population stratification could be allowed for by the genomic control method. Nineteen SNPs that were independent (>12 cM apart if on the same chromosome) and unlinked to the disease locus (*p *≥ 0.8), spread throughout the whole genome, were selected for this purpose. However, for the 300 cases and 300 controls, the estimated variance inflation factor was close to 1.00 for each of the 5 replicate samples, suggesting no significant stratification exists in this admixed population. Therefore, no adjustment for variance inflation was made for these data.

## Results

### Genome scan using model-free linkage analysis

In our analysis, we observed 8 microsatellite markers with a significance level of p ≤ 0.005 Evidence of linkage was found on chromosome 1 (22-cM region that included D01S0023 (*p *= 0.00028) and D01S0024 (*p *= 0.0016)), chromosome 3 (12-cM region that included D03S0126 (*p *= 0.0012) and D03S127 (*p *= 0.00022)), chromosome 5 (4-cM region that included D05S0172 (*p *= 0.0025)), and chromosome 9 (18-cM region that included D09S0348 (*p *= 0.000041) and D09S0349 (*p *= 0.0010)).

### Fine mapping using the WA statistic method

**Figure 1:**
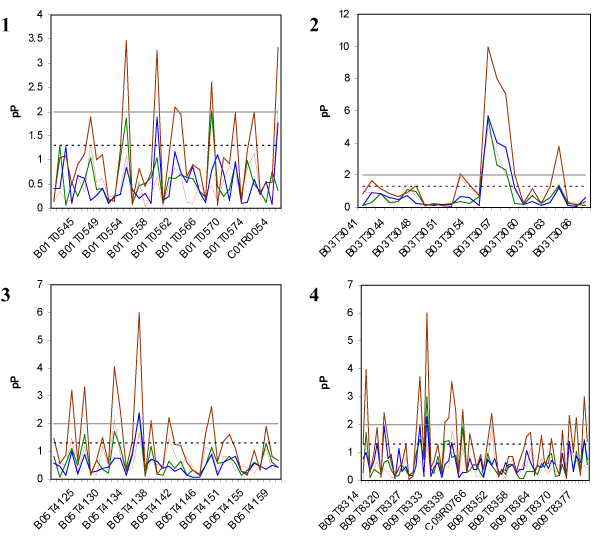
**Results of fine mapping using the WA method for chromosomes 1, 3, 5, and 9. Average of 5 replicates**. The plots show the fine mapping results using the WA method for chromosomes 1, 3, 5, and 9 in panels 1–4, respectively: blue line, Aipotu population, 100 cases and 100 controls; red line, Karangar population, 100 cases and 100 controls; green line, Danacaa population, 100 cases and 100 controls; brown line, the three samples pooled, 300 cases and 300 controls.

Genotypes for none of the 140 SNPs departed significantly from HWE at the 5% level, in either the cases or the controls. Nevertheless, we incorporated the HWD trend test to obtain a more powerful test of association. The results are shown in Figure 1, where no adjustment was made for multiple testing.

A sample size as small as 100 cases and 100 controls showed on average strong association (*p *< 0.001) with a susceptibility disease gene in the region between markers B03T3056 and B03T3058 on chromosome 3 and weaker association at marker B09T8334 on chromosome 9 (*p *< 0.05). Using the sample size of 300 cases and 300 controls, the association analysis confirmed the linkage signals in all regions on chromosomes 1, 3, 5, and 9. In this analysis we found association signals between markers B01T0553 and B01T0555 on chromosome 1 (*p *= 0.00034) (Figure 1, panel 1), markers B03T3056 and B03T3058 on chromosome 3 (*p *< 1 × 10^-10 ^) (Figure 1, panel 2), markers B05T4146 and B05T4148 on chromosome 5 (*p *= 0.00250) (Figure 1, panel 3), and markers B09T8332 and B09T8334 on chromosome 9 (*p *< 1 × 10^-6 ^) (Figure 1, panel 4).

## Discussion

In this paper we have illustrated the use of a new method for fine-mapping using a case-control design. If the mode of inheritance of a candidate gene is known, then with that knowledge a more powerful method can always be derived. The WA test was derived for a situation in which the mode of inheritance is not known. We compared the performance of the CA, WA, and HWD tests in this dataset and found that the HWD trend test always had low power. Thus, the WA maintained the advantage of the CA trend test and overcame the weakness of the HWD trend test, while the CA and WA tests had almost equal power for these particular data. It should be noted that factors such as missing data and SNP density will affect the WA test to the same extent that they will affect its components-the CA test and the HWD test.

**Figure 2:**
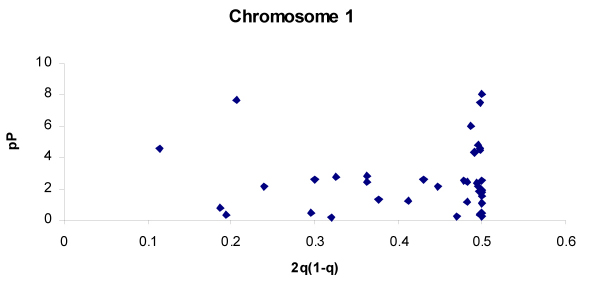
Plot of *pP *versus 2*q*(1-*q*).

Although the departure from HWE was not significant in either the cases or controls, the WA statistic uses the trend in that departure. To explain why the graphs show multiple spikes in each region on chromosomes 1, 5, and 9, we plotted *pP *= -log_10_(*p*) against the marker heterozygosity, 2*q*(1-*q*), where *q *is the marker allele frequency. Figure 2 shows the result for chromosome 1. We observe large values of *pP*, between 4 and 8, when the heterozygosity is about 0.5, i.e., when the marker is most informative. It was verified that markers with high values of *pP *but smaller values of 2*q*(1-*q*) (there are two such markers in Figure 2, heterozygosities about 0.11 and 0.20) are markers that are in high LD with those markers having heterozygosity equal to 0.5 that have high *pP *values; and similarly, those with low *pP *values but heterozygosity about 0.5, have low LD with the high *pP *markers. In other words, the strength of the signal depends on both the amount of LD and the heterozygosity of the linked marker.

## Conclusion

Using the WA statistic, a sample size as small as 100 cases and 100 controls showed on average strong association (*p *< 0.001) with a susceptibility disease gene in the region between markers B03T3056 and B03T3058 on chromosome 3 and weaker association at marker B09T8334 on chromosome 9 (*p *< 0.05). For a larger sample size (300 cases and 300 controls), as expected much more significant *pP *values were observed.

## Abbreviations

CA: Cochran-Armitage

HWD: Hardy-Weinberg disequilibrium

HWE: Hardy-Weinberg equilibrium

WA: Weighted average

## Authors' contributions

KS participated in the design of the study, performed the weighted average analysis and was involved in drafting the manuscript. MSO participated in the design of the study, performed the linkage analysis, selected the unlinked SNPs, and helped in drafting the manuscript. QL participated in the design of the study and helped in the weighted average analysis and data manipulation. RCE was involved in critical revision of the manuscript for intellectual content. All authors were involved in the interpretation of the results and approved the final manuscript.
